# Effect of temperature on interactions between soy 11S glycinin and hexanal – An off-flavour compound

**DOI:** 10.1016/j.fochms.2026.100379

**Published:** 2026-02-25

**Authors:** Cameron Ince, Lloyd Condict, John Ashton, Regine Stockmann, Stefan Kasapis

**Affiliations:** a School of Science, RMIT University, Bundoora West Campus, Plenty Road, Melbourne, VIC 3083, Australia; b Sanitarium Development and Innovation, Sanitarium Health and Wellbeing Company, Cooranbong, NSW 2265, Australia; c Commonwealth Scientific and Industrial Research Organisation (CSIRO), Agriculture and Food, 671 Sneydes Road, Private Bag 16, Werribee, VIC 3030, Australia

**Keywords:** Molecular modelling, Covalent. Bonding with heating, Plant protein, Thermal treatment, MALDI-TOF/MS, Spectroscopy

## Abstract

Thermal processing of soy proteins is known to promote interactions with lipid-derived aldehydes, yet the molecular basis and site-specificity of these reactions remain poorly defined. It is hypothesised that heat-induced structural rearrangement of soy 11S glycinin exposes discrete reactive sites within the protein, enabling preferential and potentially covalent binding of aldehyde flavour compounds. To test this, a simulated thermal treatment was performed for 20,000 ps at 353.15 K (80 °C). Following this thermal treatment and subsequent hexanal docking, a new preferential binding location nearing a lysine residue was identified, positioned within the hydrophobic core of the acidic subunit of the individual 11S chain. Traditional benchtop experiments, UV–vis spectroscopy and MALDI-TOF/MS, complemented these findings following analysis of 11S glycinin-hexanal mixtures treated at 80 °C for 60 min. MALDI-TOF/MS revealed a mass increase of approximately 84 Da, consistent with Schiff base formation between hexanal and the acidic subunit, indicating a condensation reaction rather than purely non-covalent association. Such interactions caused significant, quantifiable changes in the secondary structure of the protein determined by FTIR and CD analyses. This mechanistic insight advances understanding of flavour–protein interactions in thermally processed soy systems, aiding in the prediction of flavour retention, off-flavour formation, and protein functionality in food matrices.

## Highlights

• Conformational changes were detected upon thermal treatment of 11S soy glycinin.
• Covalent binding of hexanal to an acidic subunit of 11S was proposed (80 °C pH 7.2).
• Mass balance indicated that bond formation likely occurs *via* a Schiff base pathway.
• *In silico* analysis proposed Lys74 as the most likely site of hexanal binding.

## 1 Introduction

Soybean, an ancient staple in the south-east Asian diet tracing back thousands of years, is garnering significant traction as a plant-based alternative in the western world (Song et al., 2023). Soybeans contain all nine essential amino acids making it a serious contender in plant-based replacement against current animal-based motifs. For example, the extracted protein of soybeans (generally known as SPI) has been utilised as a key source of protein in plant-based beverage formulations. For the purposes of food safety, however, almost all milk/beverage production will undergo some form of heat treatment (Adams & Moss, 2007). This treatment presents challenges to systems containing proteins, particularly those that are thermally liable, often resulting in structural changes and/or conjugate formation that can significantly impact beverage flavour and functionality (Alrosan et al., 2024).

The soybean protein can be broken up into four molecular fractions. Two major fractions make up ∼70% (∼40% 7S vicilin and ∼ 30% 11S glycinin) and 2 minor fractions make up ∼30% (∼20% 2S conglycinin and ∼ 10% 15S). These four fractions contain excellent functional properties, which include emulsification, foaming, stabilisation and high gelling capacity (Dumitrașcu et al., 2020). Existing as a hexamer, 11S glycinin contains 5 major subunits, broken into acidic (A) and basic (B) polypeptides (A_1a_B_1b_, A_1b_B_2_, A_2_B_1a_, A_3_B_4_ and A_5_A_4_B_3_), which vary in molecular weights (∼26–44 and ∼ 14–22 kDa, respectively). These subunits are linked together *via* disulphide bonds and physical interactions forming a donut-like structure with a combined molecular mass of ∼360 kDa (Medic et al., 2014).

Soybeans are abundant in compounds that can form flavour active molecules during processing and storage (Chen et al., 2023). These include poly- and monounsaturated fatty acids. The most abundant of which are linoleic acid (omega-6), alpha-linolenic acid (omega-3) and oleic acid (omega-9) (Bukowski & Goslee, 2024). The presence of these fatty acids increases the susceptibility of soybean to the formation of malodorous compounds (Alqahtani et al., 2018). The breakdown of fatty acids into smaller molecules releases byproducts such as aldehydes through the process of oxidative degeneration or even *via* lipoxygenase activity (Shahidi & Abad, 2019). Food aroma develops from volatile compounds segregated by functional groups. Depending on their detection threshold, volatile compounds can range from those with an extremely pungent smell to undetectable as well as being favourable or malodorous (Forss, 1973).

Off-flavours appear to be a common trait of aldehyde compounds primarily developing *via* an autoxidation reaction from unsaturated lipids present within the globular membrane (Cadwallader & Singh, 2009). Hexanal (M_w_ = 100.16 g/mol) is one such aldehyde, which has caused problems in the aroma space for years, with some reports dating back to the late 70s (Takahashi et al., 1979; Wang et al., 2021). Problems with this malodorous compound is due to its relationship with oleic acid (found in large quantities within soybean), and its grassy, green and pea pod associated odour with an extremely low sensory detection threshold (0.03–0.01 ppm in water) (Forss, 1973; Tang et al., 2024). A plethora of sensory studies have reflected this, with focus group results favouring animal-based products over their plant-based counterparts largely due to the aroma imparted by hexanal (Childs et al., 2007). Despite extensive formulation and processing efforts, off-flavour development, particularly driven by hexanal, remains a major barrier to consumer acceptance of soy-based beverages, highlighting a critical gap in mechanistic understanding of protein–aldehyde behaviour during thermal processing.

As discussed in the literature, induced bio-functional modifications to the 11S glycinin molecule at the heating stage of processing is a significant challenge to current research. DSC thermograms determine that the denaturation temperature of 11S is between 90 and 92 °C making it much more thermally stable when compared to its 7S counterpart. Up until the denaturation temperature, the 11S fraction continuous to open and expand with heating (Dumitrașcu et al., 2019; Gharibzahedi et al., 2023). In doing so, new potential binding locations may be revealed (Semenova et al., 2002a, Semenova et al., 2002b). For example, Dumitrașcu et al. (2020) determined significant interactions with anthocyanins and soy proteins at around 75 °C attributed to new binding locales. However, the literature does not extensively interrogate the molecular fate of lipid-derived aldehydes within thermally treated soy protein fractions. In particular, the site-specific and potentially covalent interactions between hexanal and 11S glycinin under sub-denaturation heating conditions remain unexplored.

Given the above, it is hypothesised that heat-induced structural rearrangement of soy 11S glycinin exposes new reactive sites within the protein, thereby altering its interaction with hexanal relative to ambient conditions. Accordingly, this study aims to characterise the molecular interactions between 11S glycinin and hexanal following thermal treatment at 80 °C for 60 min, a regime selected to induce structural expansion without full subunit dissociation (Hashizume & Watanabe, 1979). Fourier transform infrared spectroscopy (FTIR) and circular dichroism (CD) were employed to probe secondary structural changes (Singh et al., 2023) along with UV–vis absorption and Matrix-Assisted Laser Desorption/Ionization – Time-of-Flight Mass Spectrometry (MALDI-TOF/MS) to determine the nature of interaction between host and ligand. Molecular simulations using Groningen Machine for Chemical Simulation (GROMACS) were further used to resolve temperature-dependent binding behaviour at the molecular level, enabling direct comparison with previously reported ambient-temperature interactions (Ince et al., 2025) and isolating the effects of heating on protein–aldehyde interactions. These findings provide mechanistic insight into heat-induced protein–aldehyde interactions in soy-based systems, informing interpretation of off-flavour behaviour during thermal processing.

## 2 Materials and methods

### 2.1 Materials

Defatted soy flour was donated by Hela Foods (Melbourne, VIC, Australia). Trizma® hydrochloride (purity >99.0%), Trizma® base (purity 99.9%), sodium bi-sulphite (SBS), hydrogen chloride (HCl) (≥ 99.0%), sodium hydroxide (NaOH) (97%), sodium chloride (NaCl) (≥ 99.5%), trifluoroacetic acid (99%) (TFA), sinapinic acid (≥ 99.0%), hexanal (analytical grade, purity >95%), glycerol (≥ 99.5%), dialysis tubes and ProteoMass™ protein MALDI-MS Calibration kit, were all purchased from Sigma-Aldrich (Sydney, NSW, Australia). Quartz cuvettes with a 10 mm pathlength were purchased from Starna Pty Ltd. (Baulkham hills, NSW, Australia). Cylindrical 1 mm quartz cell was purchased from Jasco International (Jasco International Co., Ltd., Tokyo, Japan). Ace pressure tubes (15 mL) were purchased from Ace Glass Incorporated (Vineland, NJ, United States) along with the 1000 mL centrifuge tubes provided by ThermoScientific Australia (Scoresby, VIC, Australia). All hexanal and protein stock solutions were prepared on the day of processing. Other reagents were prepared as per the provided instructions.

### 2.2 Methods

#### 2.2.1 11S extraction from soy flour

The 11S fraction was isolated from soy flour with slight modifications from existing methodology, as outlined by Smith et al. (2023). Extraction protocol included stirring for 1 h reconstituted defatted soy flour (40 g) in deionised water (600 mL) at ambient temperature and pH adjustment to 9.0 using 2 M NaOH throughout. Reconstituted samples were then centrifuged at 8198*g* for 30 min at ambient temperature using the Sorvall LYNX 6000 – ThermoScientific centrifuge with the F9-6 × 1000 LEX rotor and 1000 mL centrifuge tubes (ThermoScientific Australia, VIC, Australia). The resultant supernatant was retained and mixed in with 0.98 g/L SBS. During this step, the pH was adjusted to a pH of 4.6 using 2 M HCl for 30 min which was then stored in a fridge overnight. Mixture was centrifuged as previously, yielding the 11S pellet which underwent dialysis against Milli-Q water (Millipore Corporation, Burlington, United States) for 24 h. This process extracted the resultant 11S precipitate which was then freeze dried at −80 °C and a pressure of 10 Pa for 48 h using the Freeze Dryer-VaCo 10- Zirbus technology (Ezzi Vision) (Zirbus technology GmbH, Bad Grund (Harz), Germany) and stored in a desiccator. Purity of the 11S fraction has been confirmed with SDS-PAGE in Ince et al., 2025. Protein stock solutions were prepared gravimetrically from lyophilised 11S glycinin using an analytical balance and dissolved to a defined volume with calibrated volumetric glassware, ensuring accurate concentration determination. All working concentrations (including CD samples) were obtained by volumetric dilution from this stock.

#### 2.2.2 Sample preparation for analysis

Tris-HCL/Tris-base buffer solutions in Milli-Q water were prepared at a concentration of 10 mM and pH 7.2 following the provided manufacturer's instructions. Subsequent buffers were used to formulate/dilute stock solutions of both 11S glycinin and hexanal to the appropriate concentrations as per the designated analyses prior to heat treatment. This required the use of vacuum sealed, pressure rated tubes to prevent potential evaporation of hexanal due to its volatile nature within these experimental conditions. The pressure rated tubes (Ace Glass Incorporated, Vineland, NJ, United States) contained 8 mL of sample, which was placed in a glycerol bath, equipped with a thermowell (placed within the tubes) connected to a Testo 176 T4 datalogger (Testo SE & Co, Lenzkirch, Germany) to monitor and log the internal temperatures every 2 s. Samples were heated to 80 °C and held for 60 min to promote structural rearrangement of the protein without the extensive denaturation and precipitation associated with higher, UHT temperatures (≥135 °C for seconds). This regime approximates the cumulative thermal exposure encountered in beverage manufacturing operations, where beverages may experience extended residence at moderate temperatures (≈80 °C) during preheating and energy recovery stages prior to UHT processing (Lewis & Deeth, 2009). Then an ice water bath rapidly cooled the samples, and these were stored at −80 °C in a freezer. To remove the insoluble fractions, samples were defrosted and centrifuged for 10 min at 7400 *g* using the Sigma 1-14 k (rotor 12,094), (Sigma Laborzentrifugen Gmb, Osterode am Harz, Germany). The supernatant was retained for immediate analysis whilst the pellets were discarded.

#### 2.2.3 UV–vis measurements

UV–vis spectrophotometry was conducted using a Lambda 35 UV–vis Spectrophotometer equipped with a deuterium/halogen light source and a photomultiplier tube detector (PerkinElmer, Waltham, MA, United States) in the 200–500 nm wavelength range at a scan rate of 120 nm/min maintained at ambient conditions. Thermally treated 11S glycinin samples were diluted to 10 μM along with a combination of the protein and 150 μM hexanal. The latter was also analysed individually at a concentration of 150 μM. This protein concentration was selected based on preliminary experiments demonstrating that it provided sufficient signal and avoided detector saturation across all analytical methods employed in this study. A 1:15 M ratio of protein to hexanal was chosen to reflect the potential accumulation of lipid-derived aldehydes in food systems during oxidation. All samples were placed in a quartz cuvette with a 10 mm optical pathlength and tests were performed in triplicate (technical replicates of the same prepared sample) to ensure reproducibility.

#### 2.2.4 Circular dichroism (CD) analysis

Circular dichroism was conducted using the Jasco (J-810) spectropolarimeter (Jasco International Co., Ltd., Tokyo, Japan) under continuous nitrogen purge. Focusing in the far-UV region of 190–260 nm, a total of 3 scans were accumulated and averaged automatically by the instrument software at a scan rate of 50 nm/min and a bandwidth of 1 nm. Thermally treated 11S (10 μM), hexanal (150 μM) and a combination of the two (10 μM and 150 μM, respectively) were smeared on a cylindrical quartz cell with a path length of 0.1 mm and inspected for potential air-bubble formation. An automated coolant bath connected to a coolant-jacketed cuvette holder (RTE-111, Jasco Inc., Easton, MD, United States) ensured constant maintenance of the internal temperature at 25 °C. Acquired spectra were corrected for solvent ellipticity and were expressed as mean residue ellipticity (degrees cm^2^ dmol^−1^) using eq. (1) (Abdollahi et al., 2021).

(1) $$\left[\theta_{m}\right]=\frac{M_{w}\times 100\times \theta }{P_{r}\times C\times N}$$

where, the mean residue ellipticity is θ_m_ (degrees cm^2^ dmol^−1^), the molecular weight is M_w_ (360,000 Da for 11S) (Smith et al., 2023), the observed ellipticity is θ (degrees), the concentration of the protein is P_r_ (g/L), the pathlength of the cuvette is C (cm) and the number of 11S amino acid residues is N. Dicroweb (http://dichroweb.cryst.bbk.ac.uk; last accessed 15 March 2025) (Whitmore & Wallace, 2008) was employed to quantitively estimate the secondary structural changes of the 11S protein as a result of heat treatment and hexanal presence.

#### 2.2.5 MALDI-TOF-MS measurements

These were recorded using the AutoFlex MALDI-TOF/TOF mass spectrometer (Bruker Daltonics, Billerica, MA, United States) for thermally treated 11S (10 μM) and a combination of the protein with hexanal (10 μM and 150 μM, respectively). Samples were formulated into a solution containing a 1:1 ratio with 0.2% trifluoroacetic acid (TFA) solution, which was spotted (1 μL) onto a stainless-steel target plate in-between a dual spot (1 μL) of a saturated sinapic acid matrix (20 mg/mL in a 50% acetonitrile/0.1%TFA solution) allowing to air dry and crystallise at each step as detailed by Condict et al. (2023). A ProteoMass™ Protein MALDI-MS Calibration kit was also used to calibrate the instrument prior to each analysis. At least 3000 laser shots in the target range of 15,000–60,000 *m*/*z* accumulated at a frequency of 2000 Hz in linear positive ion detection mode was used during these analyses employing the instrument's Nd:YAG laser (355 nm).

#### 2.2.6 Fourier-transform infrared spectroscopy (FT-IR) analysis

FTIR work was carried out using the Spectrum Two Fourier transform infrared spectrometer (PerkinElmer, Norwalk, CT, United States), equipped with a GladiATR-diamond crystal attenuated total reflection device (Pike Technologies, Maddison, WI, United States), spectra were recorded using a diamond ATR crystal with a single-bounce configuration. Thermally treated 11S (10 μM), analytical grade hexanal and a combination of the two (10 μM and 150 μM, respecti*v*ely) were analysed under direct nitrogen flow. Absorbance spectra were analysed within the 4000–450 cm^−1^ region with 64 accumulated scans automatically averaged by the instrument software at a resolution of 4 cm^−1^. Using the least squares regression method, water and Tris-HCL contributions were removed to reveal the amide I and II bands (1700–1600 cm^−1^ and 1600–1500 cm^−1^, respectively). Second derivative resolution enhancement was applied using OPUS 8.2 software (Bruker Corporation, Billerica, MA, United States) to identify and quantify the amide I and II bands. The curve fitting approach utilised a Gaussian function to analyse the amide I curve. Various peaks and secondary structural components were cross-referenced with existing literature to determine the percentage of α-helical, *β*-sheet, *β*-turns and unordered structures (Barth, 2007).

#### 2.2.7 Molecular simulations and blind docking approach

To perform the molecular simulations mimicking the thermal processing conditions as outlined in this work, the GROMACS (v 2024.2) software package was utilised to perform on the RMIT Supercomputer program (RACE). An 11S chain (PDB code: 1OD5) from the crystal structure of the 11S glycinin A3B4 subunit homohexamer was downloaded from the Protein Data Bank and was utilised for the corresponding GROMACS simulations. A position restrained simulation with a total run time of 20,000 ps (ps) was performed using the ‘leap-frog’ integrator at 20 × 10^6^ steps with a time-step of 1 femtosecond (fs). The protein was centred in a cubic simulation box with a minimum solvent padding distance of 1.5 nm (15 Å) from the protein surface in all directions operating with Chemistry at Harvard Molecular Mechanics (CHARMM) and General Force Field (CGenFF) program forcefield parameters (Vanommeslaeghe et al., 2010). A sufficient amount of Na + and Cl¯ (0.0010 M) was added for neutralisation following the solvation of Transferrable intermolecular potential (TIP3P) water molecules (Jorgensen et al., 1983). The simulations were position restrained and conducted over 20 ns to examine short-timescale conformational perturbations under thermal ramping conditions rather than to capture global unfolding or long-timescale equilibrium e*v*ents.

Temperature and pressure coupling used the V-rescale and Parrinello-Rahman methods, respectively. A ‘Verlet’ cut-off scheme along with Particle Mesh Ewald for long-range electrostatics (PME) was set to 1.2 nm. A simulated annealing procedure was performed during the simulation, which consisted of a time/temperature ramp from 293 K (K) being held for 4000 ps (ps). Subsequent temperature increases of 20 K occurred e*v*ery 4000 ps until a final temperature of 353.15 K, which was also held for 4000 ps. No bond constraints were used throughout the simulation. Upon completion of the annealing simulation, trajectories of the crystal structure were analysed, with the advanced program package Visual Molecular Dynamics (VMD) (v1.9.3), to identify the secondary structural changes during the simulation as well as allowing for visualisation, determined by the resultant Root Mean Square Deviation (RMSD), and cluster analysis. The selected frames were further processed with a Bio*v*ia Discovery Studio Visualiser to develop high resolution images.

The blind docking approach was then applied for the simulated 11S glycinin chain (denoted as the host), and hexanal (denoted as the ligand). The 3D structure of hexanal was obtained from PubChem (https://pubchem.ncbi.nlm.nih.gov/compound/Hexanal). Auto Dock Vina (v 1.1.2) was employed for this work, using PyRx (v 0.8) as the graphical front end, with the resultant top ranked binding locations being visualised with the Biovia Discovery Studio Visualiser. Due to software limitations, covalent interactions were represented as a 2D image formed by MacMolPlt (v 7.7.2) along with their corresponding binding sites and binding distances (Å) (Bode & Gordon, 1998).

#### 2.2.8 Quantum mechanics calculations

As outlined in the pre*v*ious section, programs including VMD, Autodock Vina and Discovery studio contain inherent limitations when dealing with covalent interactions. As such, quantum mechanics calculations were performed to reveal potential covalent interactions which may appear within 3 Å between the host and ligand. Calculations were performed on surrounding residues (lysine, asparagine, phenylalanine, isoleucine, valine, leucine and alanine) within the vicinity of the hexanal docking location. Recreation of the potential interactions between the surrounding residues and the ligand was created in Avogadro software (v.1.2.0), which was implemented in radical mode (removing the hydrogen atoms at the appropriate positions between the ligand and amino acid) (Condict et al., 2019). Equilibration geometry calculations were then performed employing the General Atomic and Molecular Electronic Structure System (GAMESS) software package.

The type of molecules dictates the approximate optimisation method as either Restricted Hartree Fock (RHF) or Restricted Open-shell Hartree Fock (ROHF), with both methods employing an initial “Hessian” guess. Up to 99 iterations of direct Self-Consistent Field (SCF) were allowed for the calculation following a gradient convergence criterion of 0.0001 (Ha). Basis set was the 6-31G along with the B3LYP density functional theory (DFT), which was set in grid mode. Energy density plots were then analysed with MacMolPlt (v 7.7.2) and compiled in a table.

#### 2.2.9 Statistical analysis

Data were analysed by two-way ANOVA using Graph pad Prism 9.4.1 software (GraphPad software, USA) with a confidence interval of 95% (*p* ≤ 0.05), and results were expressed as mean ± standard error where appropriate.

## 3 Results and discussion

### 3.1 UV–vis and circular dichroism spectra of the 11S glycinin with hexanal complex

In the case of UV–vis, total absorption measurements take place using unpolarized or linearly polarized light, which provides information on the electronic transitions from the excited to ground state. The strength of this interaction is reflected in significant increases in absorbance, which can be seen with covalent adducts when measured against physical interactions (Czubinski & Dwiecki, 2017). This study will focus on the UV region that targets the aromatic amino acids tryptophan (Trp), tyrosine (Tyr) and phenylalanine (Phe) of 11S glycinin. These residues have absorbance peaks at approximately 280 nm, 275 nm and 257 nm, respectively. Thus, a pronounced peak at 280 nm is characteristically due to the strong absorbance provided by tryptophan (Prasad et al., 2017).

**Fig. 1:**
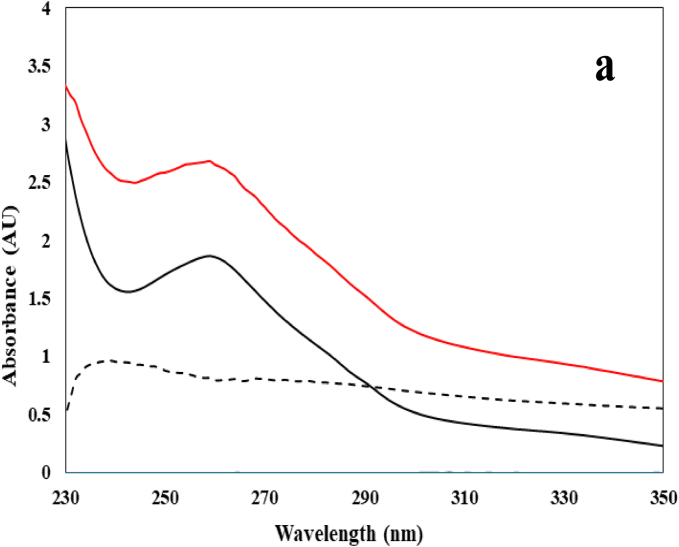

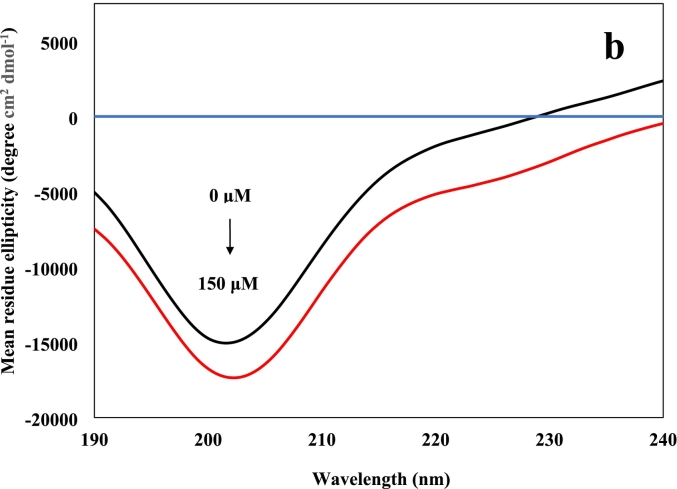
(a) UV-vis and (b) CD spectra of heat treated 11S glycinin (10 μM) (black line), 11S glycinin (10 μM) with hexanal (150 μM) (red line) and difference spectra with protein contribution removed (dashed line); hexanal (blue line) does not absorb at the bottom of the scale in UV–vis and in the middle of the scale in CD. (For interpretation of the references to colour in this figure legend, the reader is referred to the web version of this article.)

Fig. 1a displays the UV–vis spectra of 11S (10 μM), hexanal (150 μM), a combination of the two (25 μM and 150 μM, respectively) and difference spectra where the protein contribution was removed. Earlier work by Ince et al. (2025) determined that physical interactions occur between 11S soy glycinin and hexanal whilst being maintained at ambient temperature. A modest increase of 8.7% at wavelength 278 nm was identified then. Despite the absence of absorbance from hexanal, the present work shows that thermal treatment of the 11S glycinin–hexanal system results in an increase of 59.7% between 11S glycinin and hexanal. This change is consistent with temperature-induced alterations in the local environment and exposure of aromatic residues within 11S glycinin in the presence of hexanal. While UV–vis measurements in this region are not specific for hexanal adduct formation, the magnitude of the absorbance increase observed here reflects substantial changes to the environment surrounding the protein's aromatic residues, exceeding changes reported for purely physical interactions (Ince et al., 2025). These observations may therefore be consistent with the formation of covalent associations (Czubinski & Dwiecki, 2017; Liu et al., 2017) and are interpreted alongside complementary spectroscopic and mass spectrometric evidence presented herein. Similar findings were determined by Lei et al. (2022) and Abdollahi et al. (2023), with SPI being covalently linked with *β*-glucan or myricetin and *β*-lactoglobulin with ferulic acid.

Complementary to UV–vis, CD measures the absorbance difference between the left and right-handed circularly polarized light and is generally targeted in the far-UV region for secondary structural estimations (Kobayashi et al., 2011). Fig. 1b illustrates the CD spectra of heat treated 11S (10 μM) along with hexanal (150 μM) and a combination of the two components at the same levels. The spectrum of the thermally treated 11S molecule reveals a pronounced negative band between 190 and 220 nm, which is attributed to considerable random coil and *β*-sheet conformations (Mills et al., 2003). In accordance with the findings from UV–vis, hexanal provides no spectral signal. However, upon complexation with the protein, further negative readings of mean residue ellipticity (MRE) are detected from 190 to 240 nm suggesting alterations in secondary structure. These findings are similar to the interactions reported for soybean glycinin with genistein (Smith et al., 2023) and trehalose with soybean glycinin (Pengyu et al., 2021) in relation to thermal treatment. Ince et al. (2025) reported an increase in α-helix and unordered structures at the cost of the *β*-sheets and *β-*turns in soy 11S in the presence of hexanal at ambient temperature.

### 3.2 MALDI-TOF-MS analysis of the heat treated 11S glycinin and 11S glycinin-hexanal complexes

**Fig. 2:**
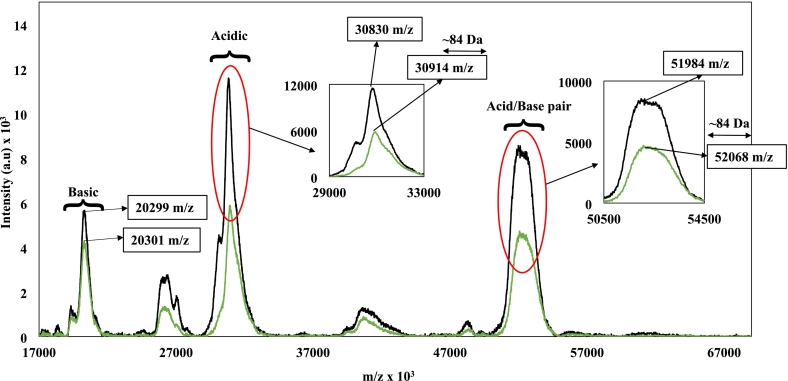
MALDI-TOF-MS spectra of heat treated 11S glycinin (10 μM, black line) and with hexanal (150 μM, green line) showing a peak shift of 84 Da indicating a covalent interaction between the acidic subunit and hexanal, which is not visible in the basic subunit. (For interpretation of the references to colour in this figure legend, the reader is referred to the web version of this article.)

Matrix-Assisted Laser Desorption/Ionization Time-of-Flight Mass Spectrometry (MALDI-TOF/MS) serves as an additional analytical technique to probe the molecular interactions between host and ligand. Due to its strong electromagnetic field, high and long-distance flight speeds as well as extreme acceleration voltage, the environment which MALDI-TOF/MS imposes only allows conditions for covalent interactions to survive (Abdollahi et al., 2023; Kafka et al., 2011). That would be visible in our case with a peak shift of the complex-combined molecular masses (potentially those of the acid and/or base subunits with the ligand). To investigate this hypothesis, MALDI-TOF/MS analysis was conducted on thermally treated 11S glycinin (10 μM) and 11S glycinin with hexanal (10 μM and 150 μM, respectively) systems shown in Fig. 2.

Three major peaks were identified at 20299, 30830 and 51,984 *m*/*z* regions with the latter attributed to the acid-base pair of 11S subunits. Individual basic and acidic subunits at 20299 and 30,830 are a result of the fracture from the acid-base pair m/z due to the extreme MALDI-TOF/MS experimental conditions (Krásný et al., 2011). At ambient temperature, hexanal does not complex covalently, as recognised by the uniformity of peaks with and without complexation (Ince et al., 2025). In Fig. 2, thermally treated samples revealed a peak shift of ∼84 Da within the acidic subunit (30,830 ➔ 30,914 m/z), which is also present within the acid-base pair of 11S subunits (51,984 ➔ 52,068 m/z). Given the limited resolving power of linear-mode MALDI-TOF for high-molecular-weight proteins, these shifts are interpreted as changes in peak centroid/maximum positions rather than definitive resolution of single-adduct mass increments. Nevertheless, with the molecular weight of hexanal being 100.16 g/mol, the peak shift of ∼84 Da is consistent with a covalent interaction through a condensation reaction (loss of one water molecule) forming a Schiff base between hexanal and the acidic subunit of 11S glycinin (Jia et al., 2023; Reineccius, 2022). Zhang et al. (2019) reported a covalent interaction between kinase inhibitor (THZ-531) and a specific cysteine residue of kinase due to a shift of the combined molecular weight.

It appears that the thermal regime utilised in this work resulted in alterations from physical to covalent interactions. Dumitrașcu et al. (2020) found cyanidin 3-glucoside and cyanidin 3-rutinoside ligands to change binding locations every 10 °C between temperatures 65 and 95 °C in their 11S and 7S host protein molecules. The same effect was reported by Feng et al. (2023) who found covalent adducts with soy protein isolate and gum arabic ligand post thermal exposure. Subjecting the 11S glycinin network to a temperature just below its denaturation temperature (∼90 °C) allows for expansion of the molecule whilst preserving the integrity of the protein structure. Whether similar interaction trends arise under shorter or milder thermal treatments may depend on thermal chemistry thresholds and the extent of protein conformational rearrangement, which remains to be systematically explored. While MALDI-TOF/MS alone cannot fully exclude non-covalent adducts or matrix-related artefacts, the observed mass shift is interpreted in the context of complementary spectroscopic, molecular simulation, and quantum mechanical analyses that collectively support covalent Schiff base formation.

### 3.3 Estimation of secondary structure changes with infrared spectroscopy

**Fig. 3:**
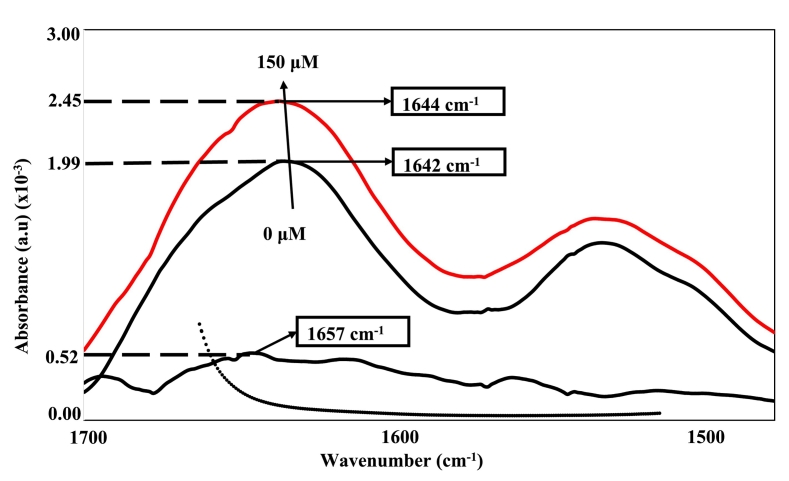
FTIR spectra in the 1700–1500 cm^−1^ region for heat treated 11S glycinin (10 μM, black line), 11S glycinin with hexanal (10 μM and 150 μM, red line), hexanal (black dotted line) and complex after subtraction of the protein contribution (black dashed dotted line). (For interpretation of the references to colour in this figure legend, the reader is referred to the web version of this article.)

Applying FTIR to the amide I region (1700–1600 cm^−1^) and amide II region (1600–1500 cm^−1^) induces vibrational modes to the protein backbone, specifically the C=O peptide stretching within the amide I region as well as C—N stretching and N—H bending vibrations within the amide II region (He et al., 2016). Fig. 3 represents the FTIR interferograms in the amide I and II regions for the thermally treated 11S glycinin (10 μM), pure hexanal (150 μM), a combination of the two (10 μM and 150 μM) and the complex after subtraction of the protein-contribution.

Of the two regions, amide I was selected due to the C=O stretching vibrations, which characteristically describe the protein-backbone secondary structural elements. Thermally treated 11S glycinin revealed a peak residing at 1642 cm^−1^. Although hexanal did not absorb in this region, its complex to the thermally processed 11S glycinin revealed an increase in absorption along with a peak shift to a higher wavelength from 1642 to 1644 cm^−1^. Upon subtraction, a positive absorption reading along with a peak shift to 1657 cm^−1^ was evident. Notably, shifts to lower wavenumbers are indicative of strengthened hydrogen bonds between complexes, as described in Wang et al. (2024). However, in this instance, the shift toward higher wavenumbers suggests a weakening of hydrogen bond strength, consistent with heat-induced conformational rearrangement of the protein backbone.

Quantification of the secondary structure was performed using OPUS software to perform curve fitting analysis within the amide I region. These findings will be compared to the secondary structure estimations from the CD spectra, with their quantification requiring use of the DICROWEB server against the CONTIN reference set. Table 1 displays the quantified secondary structural data performed from both FTIR and CD analyses. Focusing on FTIR, a significant decrease was identified for the unordered (38.5 ➔ 35.3%) and *β*-sheet (25.3 ➔ 16.4%) secondary structures upon hexanal complexation with the heated protein. In contrast, α-helical structure underwent a significant increase upon complexation nearly doubling in quantity (12.2 ➔ 23.2%). Marginal changes were identified for the *β*-turns (24.0 ➔ 25.1%), which were deemed insignificant by two-way ANOVA. Similar trends were observed by Bu et al. (2015) for lactose bound 11S molecules. The resulting structural distributions were in close agreement with values obtained independently by circular dichroism (Table 1), supporting the reliability of the FT-IR analysis. Overall, FTIR and CD analyses show consistent trends in secondary structure changes upon hexanal interaction, with minor differences likely attributable to their distinct molecular features probed by FTIR (backbone vibrations) and CD (chiral electronic transitions).

**Table 1:** Circular dichroism (CD) and Fourier-transform infrared deconvolution (FTIR) of secondary structure components for heat treated 11S glycinin and 11S glycinin with hexanal at pH 7.2.

Secondary structure component	10 μM 11S		10 μM 11S + 150 μM hexanal	
	CD	FTIR	CD	FTIR
α-helix (%)	12.5 ± 1.4	12.2 ± 0.6	21.1 ± 0.4^a^	23.2 ± 0.8^b^
*β*-sheet (%)	24.5 ± 1.7	25.3 ± 0.1	16.8 ± 0.4^a^	16.4 ± 0.5^b^
*β*-turns (%)	23.9 ± 1.0	24.0 ± 0.2	25.8 ± 0.9	25.1 ± 0.6
Unordered (%)	39.1 ± 0.2	38.5 ± 0.5	36.3 ± 1.0^a^	35.3 ± 0.9^b^

Statistical difference (*p* < 0.05) using two-way ANOVA between 11S glycinin and 11S glycinin in the presence of hexanal obtained by CD(^a^) and FTIR(^b^) measurements.

Ince et al. (2025) conducted similar work under ambient conditions with a notable difference being a much broader CD spectral band likely due to the absence of a thermal treatment. Although the α-helix increase and *β*-sheet decrease at ambient temperature are parallel tendencies with the observations in Table 1, the *β*-turns decrease and unordered increase in the absence of heating are inverse trends to the present work. The partial differences within the secondary structure suggest that the binding site may differ with heating. To further probe the effect of applied thermal treatment, annealing simulations were performed using the RMIT supercomputer program (RACE) with GROMACS.

### 3.4 Thermal treatment simulations on a 11S glycinin chain using GROMACS

When employed in combination with supercomputer intelligence, the Groningen Machine for Chemical Simulation (GROMACS) program can provide extremely accurate simulations using modelled environments to act on a predefined protein crystalline structure. Forcefield parameters, such as AMBER and CHARMM, define how atoms interact according to Newtonian mechanics, permitting the simulation to model behaviours of biological macromolecules (Yu et al., 2024). During the simulation, the crystalline structure will shuffle, change and deviate in response. The Root Mean Square Deviation (RMSD) analysis is a metric employed to probe the extent of this deviation using spatial co-ordinates x, y and z to determine its atomic positions. Deviations imposed by the simulations are measured in distance (Å) to determine whether the crystal structure has been significantly altered (Low: ∼0–2 Å and High: ∼2–5 Å) (Sapundzhi et al., 2023).

**Fig. 4:**
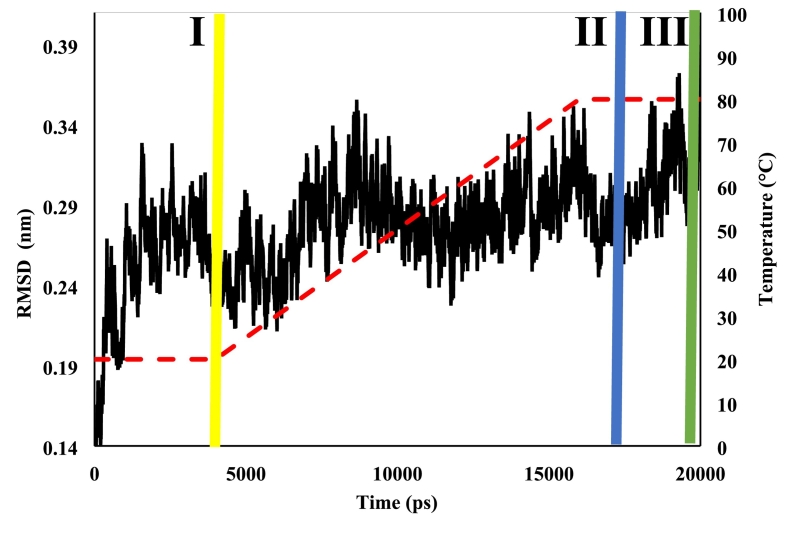
RMSD analysis of a single 11S molecule from its hexameric structure, exposed to a simulated thermal ramp for 20,000 ps depicted by the black line, with vertical lines (yellow, blue and green) corresponding to different molecular processes (red dashed line indicates temperature profile). (For interpretation of the references to colour in this figure legend, the reader is referred to the web version of this article.)

**Fig. 5:**
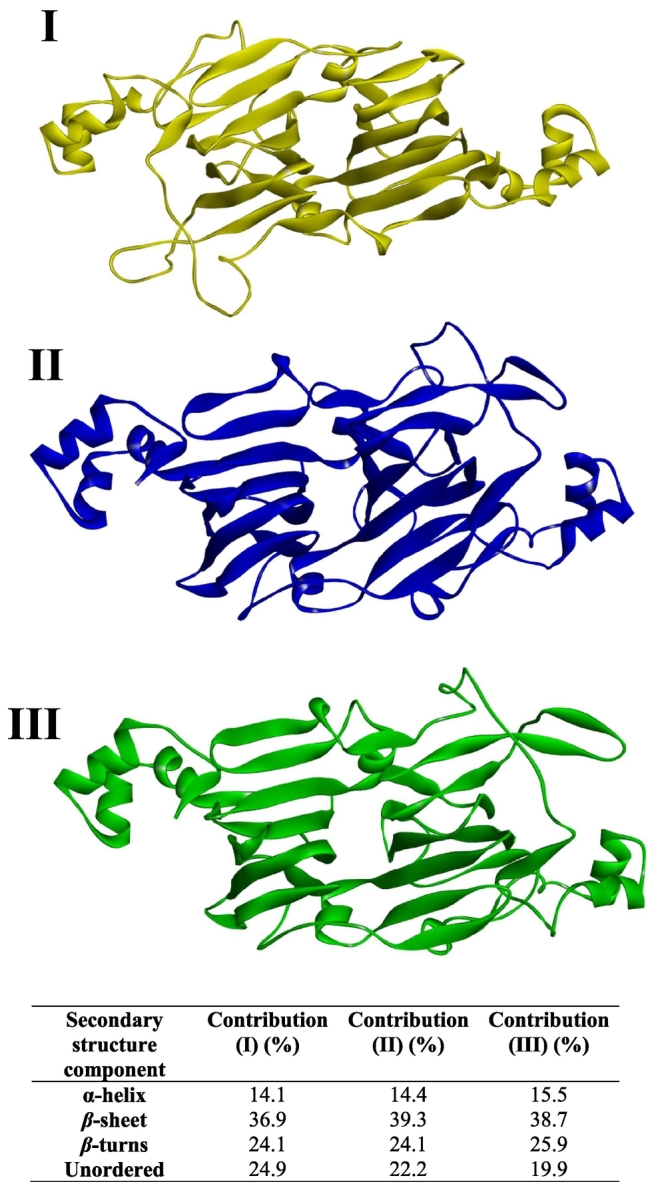
3D images of the 11S molecule at different time intervals from the RMSD analysis with colours yellow (I), blue (II) and green (III) corresponding to the different molecular processes in Fig. 4; table provides the calculated secondary structure contributions. (For interpretation of the references to colour in this figure legend, the reader is referred to the web version of this article.)

Fig. 4 displays the RMSD analysis of a single 11S chain employed from the 11S glycinin hexamer in crystal form, which has been exposed to a simulated thermal ramp. A trend was observed with an overall increase in RMSD along with sharp increases located sporadically during the analysis. These sharp increases (followed by subsequent sharp decreases) are attributed to the 11S structure opening and closing the hexameric loop, which results in secondary structural changes, as reported by Tang et al. (2024). A notable sharp drop in RMSD toward the end of the thermal ramp (from 15,000 ps to 16,500 ps) was evident and has been highlighted as an area of interest. Overall, three areas of interest were identified as vertical lines (I), (II) and (III) for specific molecular dynamics. Process (I) is the native protein in its ambient environment, process (II) is slightly beyond the end of the thermal ramp and process (III) is the protein at the conclusion of the simulation. The crystalline structure of the 11S chain at each interval was modelled and reproduced in Fig. 5, along with the corresponding secondary structure estimations, derived from the 3D crystallographic models using the VMD software, which are summarised in the adjacent table.

At ambient conditions (I), the 11S chain preserves its native structure providing secondary structural contributions of 14.1% α-helix, 36.9% *β*-sheet, 24.1% *β*-turns and 24.9% unordered structures. Fluctuations in RMSD indicate deviations of the protein conformation from the initial crystal structure during the simulation. These deviations occur coincident with the applied thermal ramp concluded in (II) and reflect increased conformational mobility and structural rearrangement of the 11S glycinin chain under the simulated conditions. *β*-sheets exhibited the greatest increase from the native state (36.9 to 39.3%) at the expense of unordered structures, which exhibited a reduction of a similar magnitude. Upon conclusion of the simulation (III), the 11S chain preserves its expansive structure just prior to entering the irreversible denaturation temperature zone (Gharibzahedi et al., 2023). Here, there is a slight reduction in *β*-sheet (38.7%) due to the loosening of certain structural elements when maintained at an elevated temperature for an extended period of time (Mills et al., 2003). It should be noted that simulations were performed on a single 11S glycinin chain; therefore, the results primarily reflect local conformational flexibility and binding site exposure at the subunit level rather than collective behaviour of the full hexamer. Furthermore, given the restrained nature and relatively short duration (20 ns) of the simulations, the observed structural deviations reflect local flexibility and thermally induced rearrangement rather than full unfolding or equilibrium conformational sampling. Feng et al. (2023) described the impact of complexation and glycated conjugation on the conformation of the soy protein molecule upon addition of gum Arabic for the development of delivery devices.

### 3.5 Molecular docking and quantum mechanics calculations

The blind docking approach was applied to the host protein at the molecular process (II), as discussed in the preceding section using a hexanal 3D structure denoted as a ligand employing AutoDock Vina (v1.1.2) and PyRx (v0.8) to identify the top ranked binding pose between the two constituents. Interacting residues within this binding locale, situated in the acidic subunit of the 11S chain, are Lys74, Asp120, Phe116, Ile68, Val70, Leu122 and Ala142 all within proximity of hexanal docked at this location. However, a key limitation with AutoDock Vina is its inability to detect covalent interactions, hence, docking results are interpreted as identifying plausible pre-reactive binding geometries rather than direct evidence of covalent interaction. To explore the feasibility of covalent modification, DFT calculations were undertaken to provide qualitative mechanistic support for covalent bond formation by comparing relative binding favourability at candidate residues. In doing so, GAMESS software was employed, to reveal the type of energy reactions (endothermic or exothermic) *via* the calculated residue bond energies. These calculations detected the presence of potential covalent interactions between the surrounding residues and hexanal in the binding pose following thermal treatment (Condict et al., 2019).

Table 2 reproduces the dissociation energies of amino acid-hexanal monomeric complexes following DFT calculations. They were determined using the disassociation energy (E_(dissoc)_), as featured in Kaur et al. (2018):

(2) $$E_{\left(\mathrm{dissoc}\right)}=\left\{E_{\left(\text{hexanal free radical}\right)}+E_{\left(\text{amino acid free radical}\right)}\right\}-E_{\left(\text{amino acid}-\text{hexanal}\right)}$$

where, E_(amino acid - hexanal)_ is the combined energy of the amino acid and hexanal ligand. E_(hexanal free radical)_ and E_(amino acid free radical)_ are the DFT energy calculations of hexanal and the respective amino acid in the free radical form, *i.e.* individual ligand and amino acid with removal of the hydrogen atom at the binding location.

**Table 2:** Dissociation energies of amino acid-hexanal monomeric complexes following density functional theory (DFT) calculations for the proposed molecular docking geometries.

**Amino acid – hexanal complex**	**E(**_**dissociation**_**)**	**Nature of dissociation**
Lys74-hexanal	1.0 х 10^3^ kJ/mol	Endothermic
Asp120-hexanal	−1.4 х 10^3^ kJ/mol	Exothermic
Phe116-hexanal	−1.9 х 10^3^ kJ/mol	Exothermic
Ile68-hexanal	−1.3 х 10^3^ kJ/mol	Exothermic
Val70-hexanal	−2.1 х 10^3^ kJ/mol	Exothermic
Leu122-hexanal	−1.4 х 10^3^ kJ/mol	Exothermic
Ala142-hexanal	−2.1 х 10^3^ kJ/mol	Exothermic
		

DFT calculations on the surrounding residues of Asp120, Phe116, Ile68, Val70, Leu122 and Ala142 were found to reflect an exothermic, that means spontaneous, dissociation reaction, which is indicative of an unfavourable covalent interaction. In contrast, Lys74-hexanal was deemed to be an endothermic (non-spontaneous) dissociation reaction with a significantly high (positive) dissociation energy (E_(dissoc)_ = 1.0 х 10^3^ kJ/mol). This demonstrates the potential for hexanal to form a stable complex with the Lys74 residue, consistent with the chemical plausibility of Schiff base formation at the ε-amino group of Lys74. In ambient conditions, interactions between 11S and hexanal are physical in nature (Ince et al., 2025), attributed to several hydrogen bonds, and by Pi-Alkyl interactions from adjacent phenylalanine residues; lysine was not detected within the vicinity of the binding location.

Lysine residues are widely considered to be suitable for covalent interactions with flavours (Reineccius, 2022). Moreover, the location of this new binding locale is situated within the acidic subunit of the 11S chain, in agreement with the earlier MALDI-TOF/MS discussion. Jia et al. (2023) reported that a condensation reaction may take place between carbonyl groups of phlorizin and ε-amino groups of lysine, which is responsible for the covalent reaction between the two constituents. Condict et al. (2019) reported covalent interactions between of *β*-casein and ferulic acid following UHT-like treatment (Michael addition) using spectroscopic, molecular docking and quantum mechanics studies.

**Fig. 6:**
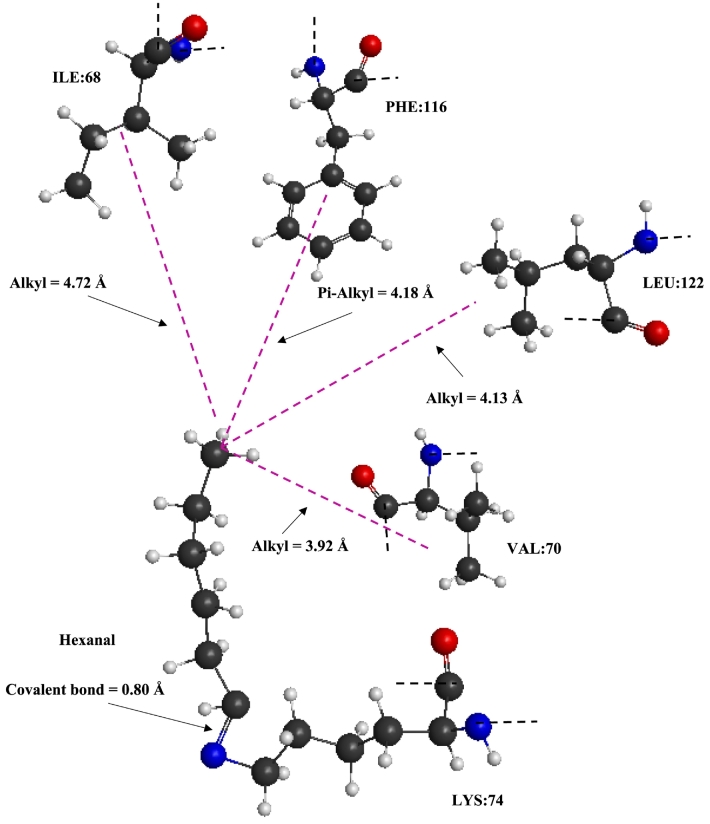
A ball-and-stick representation of the covalent linkage between LYS:74 and hexanal, as well as Pi-Alkyl and Alkyl interactions (dashed purple lines) with the surrounding residues and respective binding distances (Å); dashed black lines represent connections to the main peptide chain. (For interpretation of the references to colour in this figure legend, the reader is referred to the web version of this article.)

Fig. 6 displays a theoretical ‘ball-and-stick’ model of the potential covalent linkage between Lys74 and hexanal as the top ranked binding site following quantum mechanics calculations. Located at the terminal end of the lysine residue, hexanal forms a covalent bond with a length of 0.80 Å. Surrounding residues are located in the vicinity of the methyl end of the aldehyde being stabilised *via* alkyl bonds with the hydrophobic side chains of Val70, Leu122 and Ile68 (3.92, 4.13 and 4.72 Å respective binding distances). A Pi-Alkyl interaction with the benzene ring of Phe116 at 4.18 Å is also present. These non-covalent interactions (Alkyl and Pi-Alkyl) with the surrounding amino acids and the methyl end of hexanal provide additional stability within the binding pocket to support the covalent linkage. The new binding location was not accessible at ambient conditions for this ligand to dock, thus highlighting the effect of thermal exposure on the interaction between 11S protein and aliphatic aldehyde. Wang et al. (2022) also determined that the covalently bound heptanal ligand to myosin was further stabilised by surrounding residues *via* van der Waals forces.

## 4 Conclusions

Our findings suggest that with sufficient thermal exposure, covalent interactions form between 11S protein and hexanal. Such interactions were detected by UV–vis and MALDI-TOF/MS, with the latter analysis showing a condensation reaction with hexanal forming a Schiff base with the acidic subunit of the 11S glycinin molecule. GROMACS elucidated such findings by simulating the thermal treatment mimicking the benchtop thermal analysis to reveal that the 11S glycinin chain expands as the environmental conditions near its denaturation temperature. The top ranked binding pose revealed a location for hexanal within the hydrophobic core of the acidic subunit of the 11S molecule, interacting with a lysine residue (Lys74), a suitable amino acid for the formation of covalent interactions. The impact of the ligand on the polymeric configuration saw considerable changes in the secondary structure of the 11S protein monitored by benchtop analyses, FTIR and CD. While this work focuses on a single aldehyde–protein system under controlled thermal conditions, the findings highlight the importance of thermal history in governing flavour–protein interactions in plant protein matrices. Given the extensive use of thermal treatment within the food industry, future work should examine the generality of these observations across structurally diverse aldehydes, protein fractions, and processing conditions, as well as the functional and sensory implications of thermally induced covalent flavour binding.

## CRediT authorship contribution statement

**Cameron Ince:** Writing – original draft, Methodology, Investigation, Formal analysis. **Lloyd Condict:** Writing – review & editing, Methodology, Conceptualization. **John Ashton:** Supervision, Funding acquisition. **Regine Stockmann:** Supervision, Funding acquisition. **Stefan Kasapis:** Writing – review & editing, Supervision, Funding acquisition, Conceptualization.

## Declaration of competing interest

The authors declare that they have no known competing financial interests or personal relationships that could have appeared to influence the work reported in this paper.

## Data Availability

Data will be made available on request.
